# Oxiracetam or fastigial nucleus stimulation reduces cognitive injury at high altitude

**DOI:** 10.1002/brb3.762

**Published:** 2017-08-23

**Authors:** ShengLi Hu, JianTao Shi, Wei Xiong, WeiNa Li, LiChao Fang, Hua Feng

**Affiliations:** ^1^ Department of Neurosurgery Southwest Hospital Collaborative Innovation Center for Brain Science Third Military Medical University ChongQing China; ^2^ Department of Respiration Southwest Hospital Collaborative Innovation Center for Brain Science Third Military Medical University ChongQing China; ^3^ Department of Laboratory Medicine Southwest Hospital Collaborative Innovation Center for Brain Science Third Military Medical University ChongQing China

**Keywords:** cognitive impairment, event related potential, fastigial nucleus stimulation, high altitude, oxiracetam

## Abstract

**Background:**

Cognitive impairment is common in people travelling to high altitude. Oxiracetam and electrical stimulation of cerebellar fastigial nucleus may have beneficial impacts. This study was to investigate the effects of preconditioning with Oxiracetam or fastigial nucleus stimulation (FNS) on cognitive decline following the ascension to high altitude.

**Methods:**

The study was conducted on 60 male military voluntary members who were divided into control group, Oxiracetam group, and fastigial nucleus stimulation group. Transcranial doppler sonography, auditory evoked potential, electroencephalogram (EEG), and cognitive assessments were performed.

**Results:**

People could still suffer cognitive dysfunction at 4,000 m high altitude despite that they have lived at 1,800 m altitude for several years. The 4,000 m altitude environment also prolonged P300 and N200 latencies. Both Oxiracetam and FNS improved cognitive function, reduced the prolonged latencies of Event Related Potentials (P300 and N200), decreased the average velocity of brain arteries, and enhanced EEG power spectral entropy at 4,000 m altitude.

**Conclusions:**

Neurophysiological evidences suggest the underlying mechanism of cognitive impairments. Both Oxiracetam and FNS can reduce cognitive decline post arrival at high altitude. They could be a potential pretreatment method for cognitive dysfunction resulted from high altitude.

## 1 INTRODUCTION

There are more and more people travelling to high altitude regions for various aims including recreation, religion, business, athletic training, and military tasks in recent years. High altitude is a hypobaric hypoxic environment which could lead to a decreased oxygen supply to brain (Savourey, Launay, Besnard, Guinet, & Travers, 2003). The human brain consumes about 20% of the oxygen supplies of the whole body, which is highly vulnerable to such hypoxia (Raichle, 2010). A wide range of past studies have demonstrated that hypoxia at high altitude induces neurophysiological conditions such as dizziness, insomnia, headaches, nausea, vomiting, and fatigue, and even possibly high altitude pulmonary edema, and cerebral edema that is potentially fatal (Basnyat & Murdoch, 2003). Furthermore, it could also trigger neurocognitive dysfunction including learning, memory, language, vision, and mood (Hu, Xiong, Dai, Zhao, & Feng, 2016; Wilson, Newman, & Imray, 2009). In addition, cognitive deficits may last for some time following people's coming back to sea level from plateau (Bonnon, Noel‐Jorand, & Therme, 2000). Thus, the cognitive impairments elicited by high altitude hypoxia are of major public health importance and have economic consequences. However, to our knowledge, little is known about the effective therapeutic measures for cognitive dysfunction at high altitude.

In recent years, there is an increasing clinical application of nootropic drugs for the therapy of central nervous system diseases such as traumatic brain injury and cerebral hemorrhage. Previous evidences suggested that Oxiracetam, a new nootropic drug, improved local cerebral glucose utilization (Hokonohara, Sako, Shinoda, Tomabechi, & Yonemasu, 1992) promoted the synthesis of proteins and nucleic acids, and produced a protective effect in cerebrovascular damage (Kometani et al., 1991). Another study clearly indicated Oxiracetam inhibited cells apoptosis in brain tissues and facilitated neurological function recovery (Wang et al., 2014). Besides, recent reports demonstrated Oxiracetam improved learning and memory abilities in rats with vascular dementia (Chen, Zhou, Zhang, Feng, & Wang, 2015) and promoted cognitive impairment recovery in cerebral hypoperfusion rats (Yao, Yao, Li, Nie, & Zhang, 2016). There have been other studies suggesting that the formation of a long term memory was observed (Mondadori, Hengerer, Ducret, & Borkowski, 1994; Mondadori, Mobius, & Borkowski, 1996). Furthermore, Oxiracetam remarkably improved human's neuropsychological performance in healthy volunteers and reduced cognitive deficits in older people aged >65 years (Rozzini, Zanetti, & Bianchetti, 1993). However, whether Oxiracetam could prevent the cognitive dysfunction during exposure to high altitude is still unknown and remains to be explored.

Electrical stimulation of cerebellar fastigial nucleus is one promising approach to promote neurological functional recovery and reduce infarct volume against cerebral ischemia (Liu, Li, Li, Yu, & Li, 2012; Mandel, Talamoni Fonoff, Bor‐Seng‐Shu, Teixeira, & Chadi, 2012). Moreover, recent series of studies pointed out that fastigial nucleus stimulation (FNS) prior to and post middle cerebral artery occlusion both induced neuroprotection (Wang, Dong, Zhang, Zheng, & Wang, 2014). Another report further revealed that FNS markedly facilitated the learning and memory abilities of rats after 2 weeks or 12 weeks of recirculation following repetitive global ischemia (Tan & Yang, 2004). Taken together, FNS may have potential in treating patients with cognitive decline post arrival at high altitude.

Therefore, the purpose of this study was to investigate the effects of pretreatment with Oxiracetam or FNS on cognitive impairments following the ascension to 4,000 m altitude using cognitive assessments and auditory evoked potential (AEP), transcranial doppler sonography (TCD), and electroencephalogram (EEG) detections.

## 2 MATERIALS AND METHODS

The study was performed according to the Declaration of Helsinki and the experimental design and procedures for conducting the experiment were approved by the ethics committee on human investigation of Southwest Hospital, Third Military Medical University (the trial number is 2016‐22, registration date is 2016/03/23). Informed written consent was obtained from all the participants who volunteered for the study. Before giving written and verbal consent to participate, each volunteer was informed of the possible risk and discomforts involved in the study. They all agreed to join the study by signing the informed consent documents, which has been approved by the ethics committee.

## 3 SUBJECTS

We first offered several detailed and serious instruction‐classes to all of the subjects regarding specific concerned questions about the characteristics of high altitude and the high altitude reactions and sickness before we initiated this study. We do not use specific method to calculate the sample size. A total of 60 military participants comprising of male subjects with education of at least 12 years and between age group of 18–36 years enrolled voluntarily after being explained about the study purpose, protocol, and expected outcomes. They are lowlanders and have lived at altitude of 1,800 m for about 1–7 years and they have no any prior exposure to 4,000 m altitude prior to their participation. They all have no documented neurological disorder or history of head injury with loss of consciousness and no previous history of drug abuse, stroke, epileptic seizures. A professional medical team was employed to determine the health status and perform physical examinations of the subjects during the expedition.

### 3.1 Expedition to 4,000 m high altitude and experimental protocol

**Figure 1:**
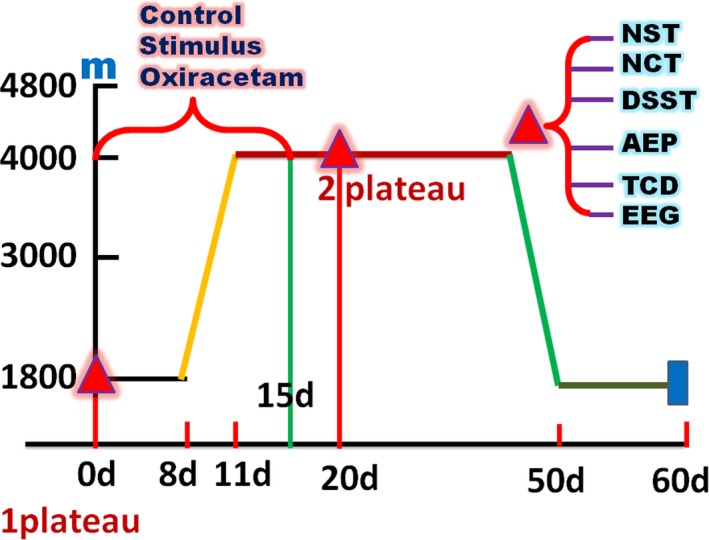
Climbing schedule of the expedition to 4,000 m high altitude and experimental procedure. The two red triangles represented the 2 time points for investigations that consisted of auditory evoked potential (AEP), transcranial Doppler (TCD), electroencephalogram (EEG), number search test (NST), number cancellation test (NCT), and digit symbol substitution test (DSST). They were performed at 1,800 m altitude and 4,000 m altitude. The treatment measures lasted for 15 days (8 days at altitude 1,800 m, 3 days on the road, and 4 days during their stay at 4,000 m high altitude)

The military volunteers lived in the places with altitude at 1,800 m. It takes them 3 days to leave from the resident place to a high altitude at 4,000 m. The first examination including AEP, TCD, and EEG accompanied by neurological cognitive tests such as number search test (NST), number cancellation test (NCT), and digit symbol substitution test (DSST) was administered at 1,800 m altitude 8 days before they leave the resident place. During the subsequent 15 days (8 days at altitude 1,800 m, 3 days on the road, and 4 days after they arrived at a 4,000 m high altitude), the subjects were divided into three groups: control group, Oxiracetam group, and stimulus group. The volunteers in the Oxiracetam group received 15 days treatments of Oxiracetam (JianLang Pharmaceutical Corp. Ltd, HuNan Province, China), 800 mg each time, 3 times per day. Subjects in the stimulus group were subjected to FNS (CVFT‐012M, RenHe electrical stimulator, ShangHai, China), which was performed according to the manufacturer's instructions using model 3 (frequency 136 Hz, electrical current 45–60 mA). Each electrical stimulation lasted for 30 min, 1 time per day, totally 15 days. In the control group, people received no any intervening measures. All volunteers were subjected to the second above examinations 9 days post arrival at the 4,000 m altitude (Figure 1).

### 3.2 Number cancellation test and number search test

Number cancellation test is a paper‐and‐pencil test, which was performed according to the methods previously described Chen et al., 2013. The test consists of a table with numerous random digits. The table was shown to the subjects. The number 3 was asked to delete. The subjects must complete the table as quickly as possible. The net score (=accurate amount−wrong amount−leaky amount/2) was recorded as the last score. All subjects were seated in a conference room and complete the test at the same time.

Number search test is also a paper‐and‐pencil test. The test consists of a table with 30 questions. Each question comprises nine random digits (one of 0–9 numbers is absent). The subjects were required to search the absent number as quickly as possible. The net score (=accurate amount−wrong amount−leaky amount/2) was recorded as the last score. All subjects were seated in a conference room and complete the test at the same time.

### 3.3 Digit symbol substitution test

Digit symbol substitution test is a paper‐and‐pencil test derived from the Wechsler Adult Intelligence Scale‐Revised. This examination measures response, attention, and processing speeds (Hu et al., 2016; Wang et al., 2013). The test consists of nine digit‐symbol pairs followed by a list of digits. Under each digit, the subject must write down the corresponding symbol as quickly as possible. In our study, the number of correct responses in 180s was recorded as the score. All subjects were seated in a conference room and took the test at the same time.

### 3.4 Auditory evoked potentials and event related potentials (ERP)

The test was performed using an auditory evoked potentials system (Keypoint 9033A07, Alpine Corp., Denmark) in an electrically shielded sound‐proof room, with the volunteers lying comfortably, with eyes closed, in order to eliminate the artifacts caused by eye movement. Electrodes were positioned as follows: reference electrode situated at Cz, recording electrode linked to right and left earlobes. The impedance between electrodes was less than 5 KΩ. The click stimulus was presented through an earphone, with alternating polarity to reduce electrical artifacts. Wave reproducibility was used to identify the presence of responses. The absolute latencies of waves I, III, V, and the values of inter‐peak intervals I–III, III–V, and I–V were measured in milliseconds (ms). The following parameters were used for the acquisition of ERP (P300 and N200): A random sequence of binaural tones (60 dB, 10 ms rise and fall, 50 ms plateau time) was presented, with a standard tone (1,000 Hz) 70% of the time and a target tone (2,000 Hz) 30% of the time. The latency of the largest positive potential occurring between 250 and 500 ms was designated the P300 component. The latency of the largest negative potential occurring between 175 and 250 ms was designated the N200 component.

### 3.5 Transcranial Doppler (TCD)

Transcranial Doppler sonography was used to noninvasively measure cerebral blood flow velocity (CBFV) in the major cerebral arteries (anterior cerebral artery, ACA; middle cerebral artery, MCA; posterior cerebral artery, PCA; vertebral artery, VA and basilar artery, BA). A TCD machine (Sonara Tek, Natus Corp., USA) with a 2‐MHz probe was used to insonate the left and right anterior, middle, and posterior cerebral arteries, and the vertebral and basilar artery. The mean CBFV (cm/s) was recorded.

### 3.6 Electroencephalogram acquisition and processing

The EEG was recorded using a portable EEG system (Xl Tek EEG32U, Natus Corp. USA) at a sampling rate of 256 Hz (band‐pass 0.3–70 Hz) from 16 leads located over left and right brain sites (Fp1, Fp2, F3, F4, C3, C4, P3, P4, O1, O2, F7, F8, T3, T4, T5, T6), using a ground lead situated at Fpz, and referenced to linked Cz. Electrodes were placed according to the universal 10–20 system and the EEG signals were recorded from electrodes placed on scalp positions viz. Impedance was maintained at less than 5 KΩ. The session commenced with an EEG recording at rest consisting of 5 min with eyes closed. Electroencephalogram power spectral entropy of representative leads was calculated. This analysis was performed with a piece of software designed by ourselves based on the technique described previously (Bachiller et al., 2014; Lu, Zhou, Zhang, Zhang, & Zheng, 2005; Zhang, Zheng, Pei, & Ouyang, 2009).

### 3.7 Statistical analysis

All data were reported as means ± *SD*. We used blinding methods to perform the data analysis. The difference value between the first examination and second examination in three groups was calculated initially. Then, statistical analysis of the difference value of each index among three groups was performed using one way analysis of variance (ANOVA) and Tukey's multiple comparison test with software SPSS 18.0. A probability value of less than .05 was considered statistically significant.

## 4 RESULTS

### 4.1 Both Oxiracetam and FNS improved cognitive function

**Figure 2:**
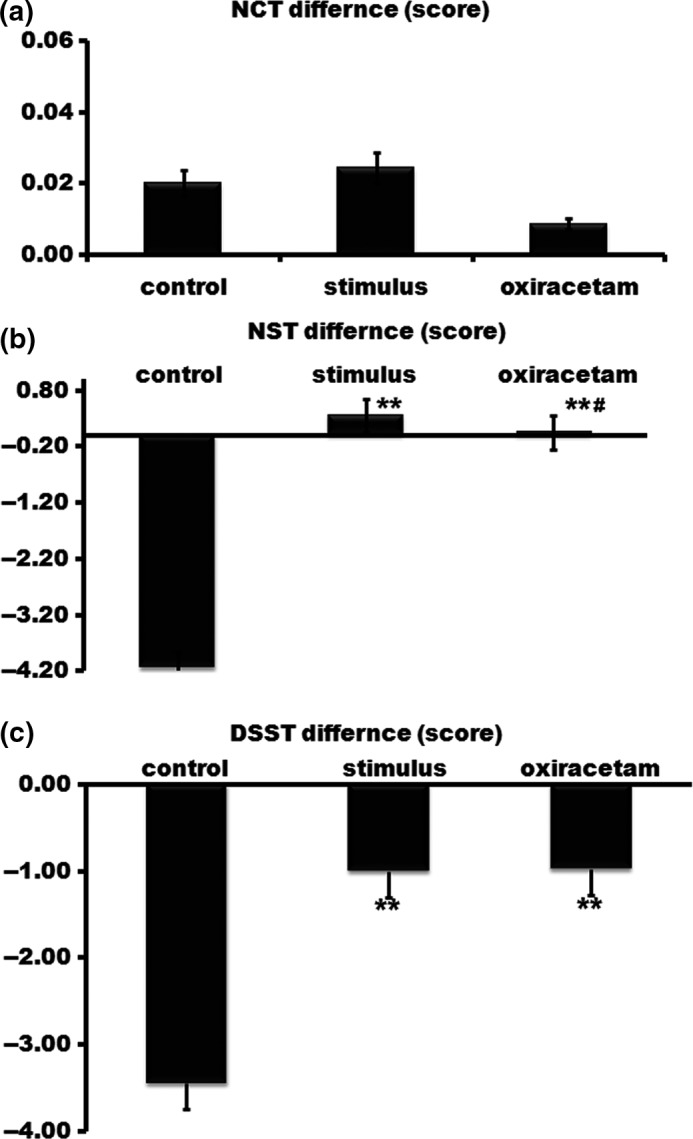
Cognitive performance was improved by both Oxiracetam and fastigial nucleus stimulation (FNS). There was no marked disparity in the number cancellation test (NCT) difference value of the two examinations among the three groups (a). The number search test (NST) (b) and digit symbol substitution test (DSST) (c) score decreased evidently in control group. However, Oxiracetam and FNS improved the NST and DSST score compared to the control group, respectively. *n* = 20, **p < .01 compared with the control group; ^#^ *p* < .05, compared with the stimulus group

We firstly explored the effects of Oxiracetam and FNS on the cognitive performance of people that went to 4,000 m high altitude from 1,800 m altitude using various rating scales including NCT, NST, and DSST. There was no significant disparity in the NCT difference value of the two examinations among the three groups. That is to say, both Oxiracetam and FNS could not promote the NCT score. The NST and DSST score decreased evidently in control group. However, Oxiracetam and FNS improved the NST and DSST score compared to the control group, respectively. There was statistical significance among the three groups (Figure 2).

### 4.2 Oxiracetam and FNS reduced the prolonged latencies of P300 and N200 at high altitude

**Figure 3:**
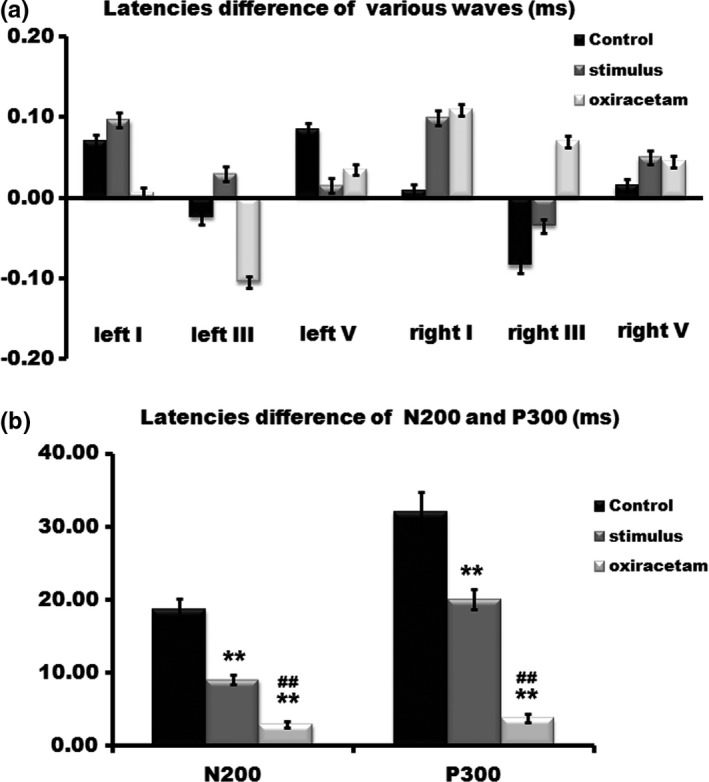
Changes in the latencies of different auditory evoked potential (AEP) waves, N200 and P300. The statistical differences in the latencies of various AEP waves among these groups could not be examined (a). The latencies of N200 and P300 were prolonged notably in the control group. Both Oxiracetam and fastigial nucleus stimulation reversed this trend. They reduced the prolonged latencies of N200 and P300 post arrival at 4,000 m high altitude (b). *n *= 20, **p < .01 compared with the control group; ^##^ *p* < .01, compared with the stimulus group

To determine the possible neurophysiological mechanism of improved cognitive function, we further detected the AEP and ERP by utilizing objective methods. We found that the latencies of P300 and N200 were prolonged notably in control group, while both Oxiracetam and FNS reversed this trend. They both reduced the prolonged latencies of ERP post arrival at the 4,000 m high altitude, which showed statistical difference compared to the control group. The latencies of various AEP waves among these groups showed no statistical differences (Figure 3).

### 4.3 Oxiracetam and FNS decreased the average velocity of brain arteries

**Figure 4:**
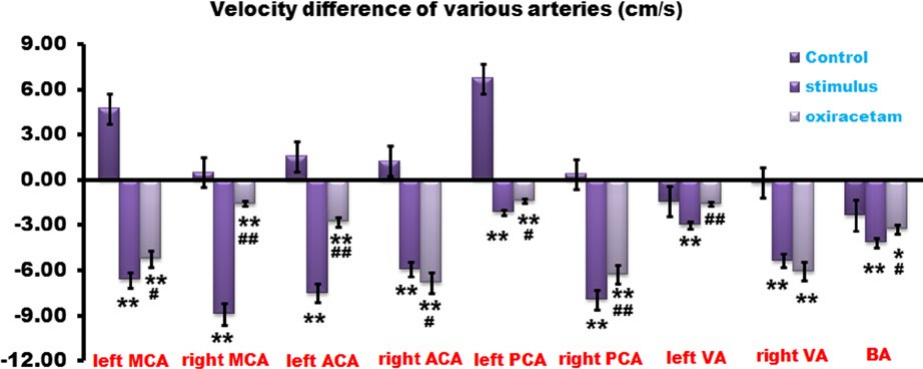
The average velocities of brain arteries were meaningfully decreased by using Oxiracetam or fastigial nucleus stimulation (FNS). TCD data revealed that velocities of anterior circulation increased while posterior circulation dropped. After Oxiracetam administration or FNS, the velocity of anterior circulation decreased evidently. At the same time, the arteries blood flow speed of posterior circulation declined much more than that in the control group. *n* = 20, *p < .05, **p < .01 compared with the control group; ^#^ *p* < .05, ^##^ *p* < .01, compared with the stimulus group

The average velocities of brain big arteries were then investigated. TCD results revealed that velocities of anterior circulation increased while posterior circulation dropped. After Oxiracetam administration or FNS, the velocities of anterior circulation decreased evidently, which were much lower than that of control group. At the same time, the arteries blood flow speed of posterior circulation declined much more than that in the control group. Statistical significance could be observed among these groups (Figure 4).

### 4.4 Oxiracetam and FNS enhanced EEG power spectral entropy

**Figure 5:**
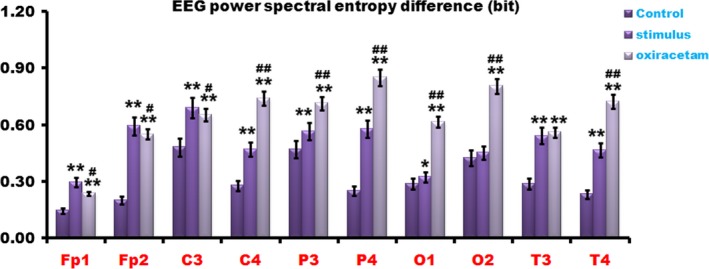
Oxiracetam and fastigial nucleus stimulation (FNS) enhanced electroencephalogram (EEG) power spectral entropy. The EEG power spectral entropy increased a little bit following arrival at 4,000 m high altitude. However, when Oxiracetam and FNS was given, the EEG power spectral entropy improved much more, which was meaningfully elevated compared with the control group. *n* = 20, *p < .05, **p < .01 compared with the control group; ^#^ *p* < .05, ^##^ *p* < .01, compared with the stimulus group

Electroencephalogram has been reported to play an important role in the cognitive function. In an attempt to ascertain the association of the EEG and cognitive performance, we further detected the EEG power spectral entropy. We observed that the EEG power spectral entropy increased a little bit following arrival at the 4,000 m high altitude. However, when Oxiracetam and FNS was given, the EEG power spectral entropy enhanced much more, which was meaningfully elevated compared with the control group (Figure 5).

## 5 DISCUSSION

The study provided convincing evidences that people could still suffer cognitive dysfunction at 4,000 m altitude despite that they have lived at 1,800 m altitude for several years. After Oxiracetam administration or FNS, the cognitive performances improved. Both Oxiracetam and FNS reduced the prolonged latencies of P300 and N200 and decreased the average velocity of brain arteries at 4,000 m high altitude. They also enhanced EEG power spectral entropy. These data clearly indicate that pretreatment with both Oxiracetam and FNS could produce cognition‐protective effects in the people who travel to the plateau regions for different objectives.

A large body of data demonstrated that people travelling to high altitudes were subjected to cognitive decline such as short‐term memory, working memory, verbal fluency, and language production (Pelamatti, Pascotto, & Semenza, 2003; Wilson et al., 2009). Moreover, a recent report suggested that the native people living in different high altitudes (3,700, 4,500, and 5,100 m) for long time showed cognitive impairment symptoms compared to human population at sea level (Zhang et al., 2013). However, whether people living at low/moderate altitude plateau (1,800 m) for years develop cognitive dysfunction following ascension to high altitude (4,000 m) has been less studied. This study indicated that the NST and DSST score decreased remarkably in control group, which suggests that people living at low/moderate altitude (1,800 m) should be given attention and care if they go to a much higher altitude for risky occupation.

AEP and ERP are often employed to assess the neurophysiological evidences for cognitive decline at high altitude. Event related potential components P300 and N200 are even considered as the indexes of sensory speed and cognitive performance, which enables direct examination of mental operations ranging from cognitive processing to performance monitoring (Hayashi, 2000). Previous studies have proved that acute exposure to a hypoxic condition via fast altitude gain or hypobaric hypoxia led to the prolonged ERP latencies, particularly for the P300 (Hayashi, Matsuzawa, Kubo, & Kobayashi, 2005; Singh et al., 2003). Our data showed that the 4,000 m high altitude also increased the latencies of P300 and N200. This further confirmed the cognitive dysfunction found through subjective rating scales.

We next found an interesting result. TCD data revealed that the mean velocities of anterior circulation increased while posterior circulation dropped. The possible reason for this is that the anterior circulation arteries may contract and the posterior circulation arteries dilate to ensure the enough blood flow of brain stem, which plays a critical role in the maintenance of basic vital sign, at expense of brain highly advanced functions. Thus, high altitude produces cognitive decline. It was further confirmed by that, after Oxiracetam administration or FNS, all the arteries dilated and the velocities of the arteries, regardless of anterior and posterior, decreased notably, which showed a statistical significance compared with the control group. Then, the cognitive function recovered.

Oxiracetam, a new ring gamma amino acid butyric acid derivative, is one kind of piracetam‐like nootropic drugs, which has been applied in neurological patients. A wide range of studies revealed that piracetam‐like drugs could protect brain against physical and chemical damages including cerebral hypoxia and improve cognitive performance (Fang et al., 2014; Uebelhack et al., 2003; Yao et al., 2016) and were beneficial to patients with senile dementia (Nicholson, 1990). Hence, we explored the effects of Oxiracetam on cognitive performance in people travelling to high altitude from 1,800 m altitude. We found that Oxiracetam improved cognitive function, which may result from the vasodilation of the anterior circulation and elevation of EEG power spectral entropy. The effects of Oxiracetam on cognitive improvement may involve many possible mechanisms. Firstly, it may relate to neurotransmission. Hlinak et al. reported that Oxiracetam could alleviate *N*methyl‐d‐aspartic acid (NMDA) antagonist MK‐801 induced long term memory damage (Hlinak & Krejci, 2000). It has also been found that Oxiracetam forestalled memory deficits elicited by scopolamine but not the diazepam (Hlinak & Krejci, 2002). A recent study suggested Oxiracetam could improve long term potentiation (LTP) caused by chronic cerebral hypoperfusion (Yao et al., 2016). It is common that LTP is associated strongly with learning and memory abilities and mainly depends on binding of excitatory receptors, such as NMDA receptor, and their neurotransmitter (Lynch, 2004; Zarnowska et al., 2015). Hence, Oxiracetam may potentiate neurotransmission, impact ion fluxes, enhance LTP, and prevent cognitive impairment. In addition, Oxiracetam could increase brain metabolism and promote the synthesis of functional molecular synthesis including neurotransmitter and excitatory receptors, which is beneficial to cognitive improvement. Yao et al. (2016) also found that Oxiracetam enhanced the levels of brain derivative growth factor and it was considered to be closely associated with LTP and cognitive function in recent years (Montalbano, Baj, Papadia, Tongiorgi, & Sciancalepore, 2013; Santos et al., 2015; Schildt, Endres, Lessmann, & Edelmann, 2013). Last but not least, Oxiracetam influenced the morphometry of astrocytes and increased the level of high energy phosphates (Gabryel, Trzeciak, Pudelko, & Cieslik, 1999). So it may improve cognitive performance through astrocyte's supportive function.

Situated at the top of the fourth ventricle, the fastigial nucleus (FN) consists of adrenergic intrinsic neurons and nerve fibers. Electrical current could stimulate the fibers passing through the FN, which leads to vasodilation and increase in cerebral blood flow (Wang et al., 2014). Thus, FNS possesses the potential to produce neuroprotective and cognition‐preserving effects. The present study showed that cognitive function recovered, prolonged ERP latencies shortened, and EEG power spectral entropy enhanced following FNS, which reflects indirectly the extent of excitement of brain (Zhang et al., 2009). These data indicated FNS may be a potential pretreatment method for cognitive decline resulted from high altitude.

This study, to the best of our knowledge, is the first report on the application of Oxiracetam and FNS in human prior to high altitude. Our findings demonstrated that the extensively utilized measures in clinical practice, Oxiracetam and FNS, could exert a major role in the prevention of cognitive impairments. Because the cognitive function is very important to people who go to plateau for work which may lead to risks due to cognitive damage, this study could be of considerable clinical relevance for occupational health at high altitude and has important public health significances with the increasing number of people going to high altitude.

## 6 CONCLUSIONS

Cognitive dysfunction occurs in people travelling to 4,000 m high altitude in spite of their living at 1,800 m altitude for years. Both Oxiracetam and FNS can reduce such cognitive impairments. They could be a potential preconditioning approach for cognitive decline induced by high altitude. A shortage of this study is that the underlying mechanism is unknown and further research is needed in future work.

## CONFLICT OF INTEREST

This work has no conflict of interest to declare.
